# Mother’s Milk: A Purposeful Contribution to the Development of the Infant Microbiota and Immunity

**DOI:** 10.3389/fimmu.2018.00361

**Published:** 2018-02-28

**Authors:** Kirsty Le Doare, Beth Holder, Aisha Bassett, Pia S. Pannaraj

**Affiliations:** ^1^Centre for International Child Health, Imperial College London, London, United Kingdom; ^2^Paediatrics, Imperial College London, London, United Kingdom; ^3^Paediatric Infectious Diseases Research Group, St. George’s, University of London, London, United Kingdom; ^4^Vaccines & Immunity Theme, MRC Unit The Gambia, Fajara, Gambia; ^5^Division of Infectious Diseases, Children’s Hospital Los Angeles, Los Angeles, CA, United States; ^6^Department of Pediatrics and Molecular Microbiology and Immunology, University of Southern California, Los Angeles, CA, United States

**Keywords:** breast milk, microbiota, microbiome, human milk oligosaccharides, exosomes, extracellular vesicles, infant microbiome, breast milk microbiome

## Abstract

Breast milk is the perfect nutrition for infants, a result of millions of years of evolution. In addition to providing a source of nutrition, breast milk contains a diverse array of microbiota and myriad biologically active components that are thought to guide the infant’s developing mucosal immune system. It is believed that bacteria from the mother’s intestine may translocate to breast milk and dynamically transfer to the infant. Such interplay between mother and her infant is a key to establishing a healthy infant intestinal microbiome. These intestinal bacteria protect against many respiratory and diarrheal illnesses, but are subject to environmental stresses such as antibiotic use. Orchestrating the development of the microbiota are the human milk oligosaccharides (HMOs), the synthesis of which are partially determined by the maternal genotype. HMOs are thought to play a role in preventing pathogenic bacterial adhesion though multiple mechanisms, while also providing nutrition for the microbiome. Extracellular vesicles (EVs), including exosomes, carry a diverse cargo, including mRNA, miRNA, and cytosolic and membrane-bound proteins, and are readily detectable in human breast milk. Strongly implicated in cell–cell signaling, EVs could therefore may play a further role in the development of the infant microbiome. This review considers the emerging role of breast milk microbiota, bioactive HMOs, and EVs in the establishment of the neonatal microbiome and the consequent potential for modulation of neonatal immune system development.

## Introduction

Breastfeeding confers protection against respiratory and gastrointestinal infections and is associated with a reduced risk of inflammatory diseases such as asthma, atopy, diabetes, obesity, and inflammatory bowel disease (1–7). Prolonged and exclusively breastfed infants have improved cognitive development (8, 9). Human milk continues the transfer of immunity from mother to child that started *in utero*, providing a nurturing environment that protects against infection and develops the infant intestinal mucosa, microbiota, and their own immunologic defenses. Breast milk is a specialized secretion in which immune response is highly targeted against microorganisms in the mother’s gut and airway, providing an important defense against the same pathogens likely encountered by her infant (10). More recent studies suggest that breast milk not only provides passive protection but also directly modulates the immunological development of the breastfed infant through a variety of personalized microbial and immune factors transmitted from mother to child (11–14). These early imprinting events are crucial for immunologic and metabolic homeostasis.

Breast milk immune factors are at their highest concentrations in the colostrum (15), suggesting an immunologic function of milk when the infant is at highest risk of exposure to new pathogens. However, they continue to be dynamically present throughout the lactation period. Bioactive factors transferred to the infant *via* breastfeeding including immunoglobulins, cytokines, chemokines, growth factors, hormones, and lactoferrin have been reviewed in detail elsewhere (15–17). This review will focus on the roles of breast milk microbiota in the establishment of the infant intestinal microbiota, human milk oligosaccharides (HMOs) in shaping the microbiota, and extracellular vesicles (EVs) in modulation of the host–microbe interactions. Breast milk microbiota, HMOs, and EVs are emerging as areas of potential therapeutic interests due to their implications for infant immune development, health, and scope for therapeutic manipulation.

## Breast Milk Microbiota

Breast milk comprises several hundred bacterial species and harbors bacteria at concentrations of approximately 1,000 colony-forming units (CFUs)/mL (18, 19). It is estimated that breastfed infants ingest up to 800,000 bacteria daily (20). Following a dose of microbes at birth (21), breast milk is the immediate next fundamental source of microbes seeding the infant’s gut (22, 23). Many epidemiologic studies have documented differences in the composition of gut microbiota in breastfed and formula-fed infants (24–26). Human milk directly contributes to the establishment of the infant intestinal microbiome (19, 20, 23, 27–29). Multiple studies have documented the sharing of specific microbial strains of *Bifidobacterium, Lactobacillus, Enterococcus*, and *Staphylococcus* species between breast milk and infant stool (30–32). During the first month of life, infants who primarily breastfeed share 28% of their stool microbes with their mother’s milk microbes. The frequency of shared microbes increases with the proportion of daily breast milk intake in a dose-dependent manner (23). These findings strongly suggest the transfer of microbes from breast milk to the infant gut. Although an interindividual variation in the types and abundance of different bacteria in human milk exists, the bacteria found in the infant gut most resemble the bacteria from their own mother (23).

While early studies employed culture-dependent methods, recent development of culture-independent techniques, such as next-generation sequencing, has expanded our understanding of the composition and diversity of the breast milk microbiome (33–35). *Streptococcus* and *Staphylococcus* species are the most commonly identified bacterial families in human milk, followed by *Bifidobacterium, Lactobacillus, Propionibacteria, Enterococcus*, and members of the *Enterobacteriaceae* family (23, 28, 35, 36). Several hundred bacterial species have been identified with higher diversity in colostrum compared to transition and mature milk (18).

The origin of bacteria in breast milk is not well established. Breast tissue itself contains a diverse population of bacteria (37). A dynamic cycling of bacteria between mother and infant with retrograde flow from maternal commensal skin flora to infant mouth flora during breastfeeding (38) likely contributes to the bacterial communities (39). However, commensal contamination does not fully account for the diversity of human milk microbes or the presence of strictly anaerobic species such as *Bifidobacterium, Clostridium*, or *Bacteroides* species. Milk microbial community composition has been shown to differ from communities on the surrounding areolar skin and infant mouth (35, 40). Another proposed theory is an enteromammary pathway whereby maternal intestinal bacteria migrate to the mammary glands *via* an endogenous cellular route during pregnancy and lactation (19, 28, 41). It has been hypothesized that bacteria first translocate the maternal gut by internalization in dendritic cells and then circulate to the mammary gland *via* the lymphatic and blood circulation (42). This specialized form of mother–infant communication of transferring microbes from the mother’s gut to the infant *via* breastfeeding needs further investigation.

**Figure 1:**
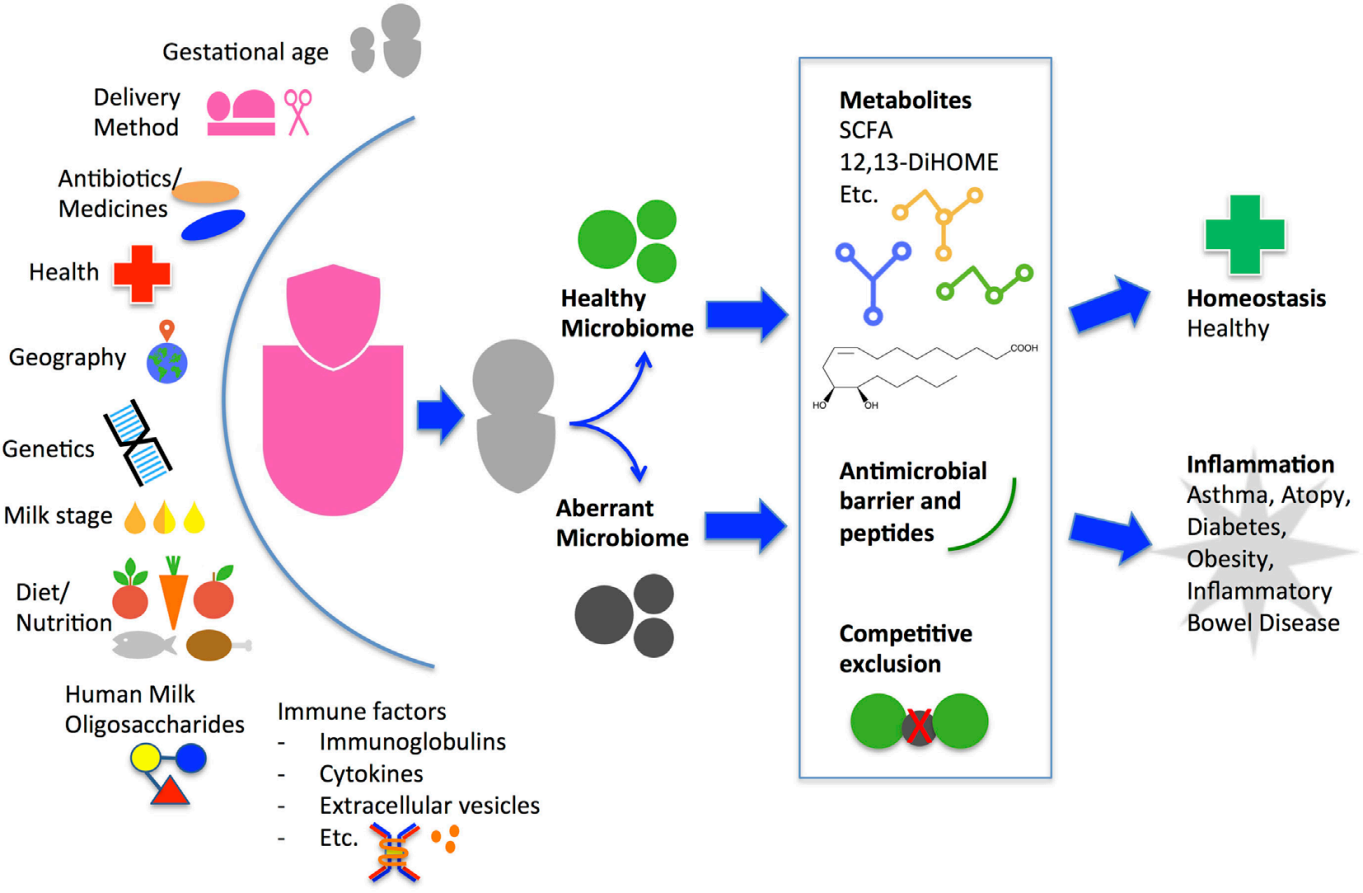
Factors that influence maternal breast milk microbiome and proposed mechanism of how breast milk may alter the infant gut microbiome and health outcome. A myriad of environmental, genetic, and immune factors personalize a mother’s milk for delivery to her infant. Starting from the initial feeding, the breast milk microbes and human milk oligosaccharides contribute to the composition and diversity of the infant gut microbiome. The initial gut microbes may continue to promote colonization of a healthy community or an aberrant community. During the critical window of immune development, the community types may induce metabolic alterations leading to differing immune phenotypes and long-term health outcomes. SCFA, short-chain fatty acids.

Maternal factors affect milk microbiota composition and diversity (Figure 1). Higher diversity has been reported in milk from mothers who deliver vaginally compared with C-section by some groups (18, 43, 44) but not others (23, 45). Milk bacterial profiles do not significantly differ in relation to maternal age, infant gender, or race/ethnicity within a geographical region but do differ across geographic locations of Europe, Africa, and Asia (23, 45, 46). *Bifidobacterium* species concentration was higher in term deliveries than preterm deliveries (44). Total bacteria concentration using quantitative PCR is lower in colostrum than in transitional and mature milk, with increasing levels of *Bifidobacterium* and *Enterococcus* species over time (18, 44). Maternal health alters milk microbiota composition and diversity as evidenced by comparative studies of healthy mothers to those with obesity, celiac disease, and human immunodeficiency virus (HIV) (18, 47, 48). Immunomodulatory cytokines secreted in breast milk from healthy women such as transforming growth factor beta (TGFβ) 1 and TGFβ2 are associated with increased early-life microbial richness, evenness, diversity, and increased abundance of taxa protective against atopic diseases (49). Unsurprisingly, maternal antibiotic use and chemotherapy decrease bacterial diversity in breast milk (50, 51); how this impacts on the infant microbiome and immune system development in the long term is currently unknown. More studies are warranted to understand how maternal genetics, culture, environment, nutritional status, and inflammatory states from acute or chronic diseases affect breast milk microbiota.

### Role of Breast Milk Microbiota in the Infant Gut

Breast milk bacteria have both immediate- and long-term roles in reducing the incidence and severity of bacterial infections in breastfed infants by multiple mechanisms. Commensal bacteria can competitively exclude or express antimicrobial properties against pathogenic bacteria. For example, *Lactobacilli* isolated from breast milk have been shown to inhibit adhesion and growth of gastrointestinal pathogens, including *Escherichia coli, Shigella* spp, *Pseudomonas* spp, and *Salmonella* spp strains (52–54). Five breast milk *Lactobacilli* strains increased mucin gene expression by intestinal enterocytes to form an antibacterial barrier (53). Administration of a breast milk *Lactobacilli* strain in a double-blind controlled trial to infants 6–12 months of age reduced the incidence of gastrointestinal, respiratory, and total infections by 46, 27, and 30%, respectively (55). The significant increase in bacterial counts of *Lactobacilli* and bifidobacteria in the experimental group compared with the controls was thought to explain the reduced clinical infection episodes although the pathogenic bacteria counts were not measured. Another study found that 30% of human milk contains nisin-producing bacteria that can survive passage through the intestine (56). Nisin is a bacteriocin used by the dairy industry to prevent spore germination and inhibit *Clostridium botulinum* and *Bacillus cereus*. *Staphylococcus epidermidis* and *Streptococcus salivarius* from expressed breast milk also possesses antimicrobial activity against pathogenic *Staphylococcus aureus* (20). Although there are many studies of antimicrobial peptides and molecules in the intestine, more studies are necessary to understand the specific antimicrobial activities of breast milk bacteria.

Increasing evidence in animals points to the instrumental role of microbiota in the development and instruction of the immune system (57, 58). In the absence of intestinal bacteria, animals have defects in lymphoid tissue development within the spleen, thymus, and lymph nodes. Germ-free intestines have reduced numbers of lamina propria CD4+ cells, IgA-producing cells, and hypoplastic Peyer’s patches (59). Germ-free mice typically are Th2 skewed but achieve a balance of Th1/Th2 cytokine production after the introduction of symbiotic bacteria (60). Breast milk *Lactobacillus* strains have been shown to enhance macrophage production of Th1 cytokines including Il-2, IL-12, and TNF-alpha (61). An early human study has suggested better Th1 responses in breastfed children compared to formula-fed children with immunomodulating effects lasting beyond weaning (62). Another *in vitro* study showed that *Lactobacillus fermentum* and *Lactobacillus salivarius* were potent activators of natural killer cells affecting innate immunity as well as moderate activators of CD4+ and CD8+ T cells and regulator T cells affecting acquired immunity (63). Breastfed rhesus macaque infants develop distinct gut microbiota and robust populations of memory T cells and T helper 17 cells compared to bottle-fed infants (64). Whether these mechanisms also exists in humans is not yet known.

### Critical Window of Opportunity for Immune Effects

The World Health Organization recommends exclusive breastfeeding during the first 6 months of life (65). This time period of exclusive milk ingestion is also a critical window for microbial imprinting (23, 66, 67). The infant microbiome comprises a dynamic community of bacteria that transforms throughout infancy and into early childhood, but the community assembly is non-random and depends on early-life events (57, 66). Dysbiosis during this critical developmental window during a time of exclusive milk ingestion may have long-term health implications (57, 68). Germ-free mice have an overaccumulation of invariant natural killer (iNKT) cells leading to susceptibility to colitis, but colonization with standard microbiota before 2 weeks of life but not after, normalizes iNKT cell numbers and protected against colitis (69). Similarly, germ-free adult mice have elevated serum IgE levels associated with exaggerated allergic responses, but mice colonized with standard microbiota before 4 weeks of age, but not after, have normal IgE levels (70). Oral administration of *Bifidobacterium breve* in mice induces proliferation of FoxP3+ regulatory T cells, but only if administered during the pre-weaning stage (54). Even transient perturbations in the microbiota in early life with penicillin is sufficient to induce sustained metabolic alterations and changes in the expression of immune genes in mice (68). Longitudinal human cohorts have supported the long-term implications of early dysbiosis. Arrieta et al. showed transient gut dysbiosis during the first 100 days of life put infants at higher risk for asthma (71). The relative abundance of *Lachnospira, Veillonella, Faecalibacterium*, and *Rothia* was significantly lower in children at risk of asthma. These genera are present in breast milk (23, 36). Fujimura et al. found a microbiota conformation that was significantly associated with a higher risk of atopy; the conformation was only detectable in children younger than 6 months. By using fecal water from these infants cultured *ex vivo* with human adult peripheral T cells, the investigators showed enhanced induction of IL4+ CD4+ T cells and decreased abundance of CD4+ CD25+ FOXP3+ cells, suggesting that dysbiosis promotes CD4+ T cell dysfunction associated with atopy (72). The progressive establishment of the infant microbiota is vital for educating their immune system to tolerance and reactivity to maintain health throughout life. A recent study by Bäckhed et al. suggests that cessation of breastfeeding rather than introduction of solid foods is the major driver in the development of an adult microbiota (73). Indeed, Ding and Schloss found that history of breastfeeding as an infant dictated bacterial community composition as adults (74).

### Breast Milk Virome

Viruses are also known to be transmitted through breast milk (75) and likely contribute to the gut ecology of the developing infant. The assembly of phage and eukaryotic components of the infant gut virome is affected by health and nutritional status (76). Breitbart et al. did not find similar viral sequences in maternal breast milk and the infant stool in their one infant followed over time (77). However, a recent study of 25 mother–infant pairs identified bifidobacterial communities and bifidophages in maternal milk and infant stool, strongly suggesting vertical transmission through breastfeeding (78). Because the majority of viruses inhabiting the infant and adult gut are bacteriophages (77, 79), they have the ability to kill bacteria or provide them with potentially beneficial gene functions to shape the bacterial community and long-term health. Longitudinal studies to determine the role of breastfeeding in the establishment of the infant gut virome and the viral–bacterial interactions are warranted.

## Human Milk Oligosaccharides

**Figure 2:**
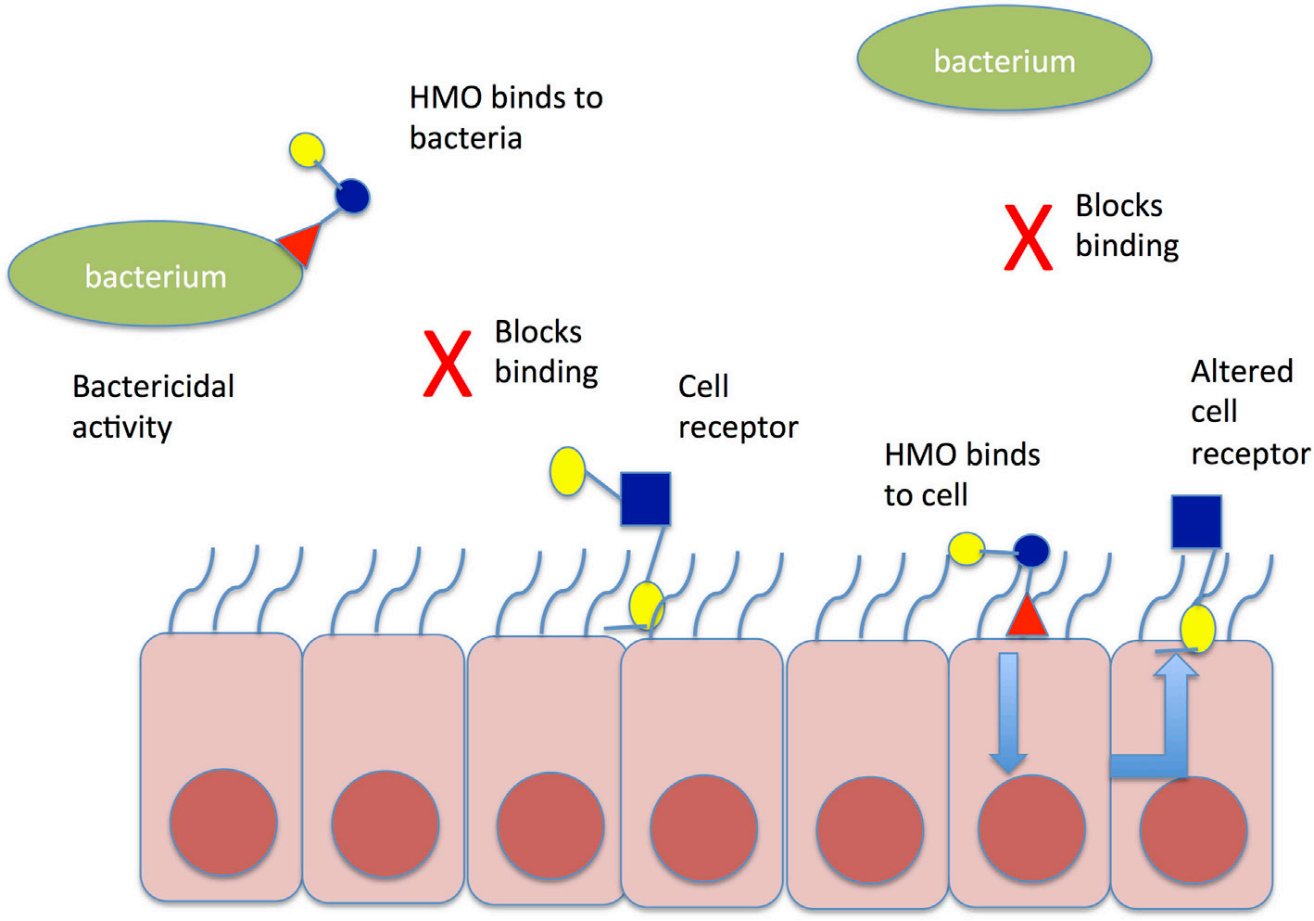
Mechanism of action of HMO to prevent aberrant pathogen colonization. HMO may bind directly to bacteria in the gut lumen causing conformational change in bacterial binding sites and preventing binding to cell receptors; alternatively, HMO may bind directly to gut epithelial cells causing altered expression of cell receptors, which prevent pathogen binding to gut epithelial cells. HMO, human milk oligosaccharide.

Human milk oligosaccharides (HMO) may further influence the establishment of a healthy microbiome, by binding potentially harmful bacteria in the intestinal lumen, asserting direct antimicrobial effects, modulating the intestinal epithelial cell immune response, and thereby promoting the growth of “good bacteria” (Figure 2). HMOs are soluble complex carbohydrates that are synthesized in the mammary glands dependent on maternal genotype, including the genes that determine the Lewis blood group antigen.

HMO are indigestible by the infant. Instead, they function as prebiotics, encouraging the growth of certain strains of beneficial bacteria, such as *Bifidobacterium infantis*, within the infant gastrointestinal tract (80), thus preventing infection by allowing the microbiota to outcompete potential pathogenic organisms (81, 82). Once ingested by the infant, HMOs are thought to inhibit the adherence of pathogens to the intestinal epithelium by acting as a decoy receptor for pathogens, which prevents attachment to host cells, thereby preventing pathogen adhesion and invasion (83). HMOs are also thought to have direct antimicrobial effects on certain pathogens (81). Finally, HMOs have been observed to modulate intestinal epithelial cell responses, as well as act as immune modulators. HMOs alter the environment of the intestine, by reducing cell growth, and inducing differentiation and apoptosis (84). They alter immune responses by shifting T cell responses to a balanced Th1/Th2 cytokine production (85).

Genetic differences are responsible for differences in HMO profiles in breast milk (86–89), although HMO abundance changes throughout lactation. Therefore, mothers possessing different genotypes, and thus different HMO profiles, may protect their infants against certain infections to a greater or lesser extent, depending on the presence of specific HMOs. Likewise, the different HMOs produced alter the types of microbiota colonizing infants, as well as the timing of the establishment of the microbiota (90). Because of their complexity, no human milk identical HMOs have been synthesized. However, non-human milk-derived alternatives that may have similar bioactive properties are gaining interest. In a recent placebo-controlled trial of 4,556 infants from India, a plant oligosaccharide, fructooligosaccharide, was given to infants together with *Lactobacillus plantarum* and demonstrated a reduced risk of sepsis and death in those in the treatment arm (RR, 0.6; CI, 0.48–0.74) compared to those in the control arm (91). The results highlight a potential role for HMOs and non-milk oligosaccharides in preventing neonatal infection.

HMO are thought to play an important role in preventing neonatal diarrheal and respiratory tract infections (92, 93). Several HMOs have been implicated in protection against bacterial and viral infections in neonates, including fucosyltransferase enzyme (FUT3), associated with the Lewis–Secretor gene (89) and 2′-fucosyllactose (2′-FL), associated with the Secretor gene (FUT2) (87). High concentrations of 2′-FL are associated with reduced risk of infant *Campylobacter jejuni* (94) and rotavirus infections (95). However, it has been noted that there is a rotavirus strain-specific effect of different HMOs, both alone and in combination (95, 96). Lewis–secretor positive infants in Burkina Faso and Nicaragua appear to have increased susceptibility to rotavirus infection compared to Lewis-negative infants. As the Lewis antigen is partially responsible for HMO abundance, this finding may explain the reduced efficacy of the live oral rotavirus vaccine in Africa where the majority of women are Lewis–Secretor negative (96). Conversely, an observational study undertaken in the United States found severe rotavirus gastroenteritis to be essentially absent in children who had a genetic polymorphism that inactivates FUT2 expression on the intestinal epithelium, which may indicate further strain-specific adaptations of HMOs (97). Infants who received milk containing a low concentration of lacto-*N*-difucohexaose have an increased incidence of calicivirus diarrhea (98). Other HMO combinations in breast milk have also been associated with reduced risk of HIV transmission in Zambia (99).

It has been suggested that HMOs could be used therapeutically to harness these antibiotic benefits together with standard antibiotics (100, 101). Research to date has primarily focused on developing such adjuncts by investigating antiadhesive properties of HMOs *in vitro*. These include the ability of HMOs to reduce *Streptococcus pneumoniae* adherence to cells of the oropharynx (102) and gastrointestinal adherence with *Escherichia coli* (103–105). Specific HMOs such as FUT3 have been implicated in increased killing of Group B *Streptococcus* (GBS) *in vitro* (106–108). The Bode laboratories have determined that GBS requires specific HMO to proliferate *in vitro* (101), Further *in vitro* investigation revealed that GBS uses a glycosyltransferase, which incorporates HMOs into the cell membrane, preventing bacterial proliferation. The Townsend and Le Doare laboratories have also identified Lewis–Secretor status to be important in reducing biofilm associated with GBS (106, 107). Further studies have identified that HMO-2′-FL also acts as a decoy receptor for norovirus (109). Animal models also report increased Th1 responses against RSV in mice given a prebiotic containing HMOs (110). HMOs are emerging as a novel potential adjunct to antibiotic therapy, but there is much uncertainty as to individual HMO function and synthesizing individual HMOs in the laboratory for use in clinical trials has proven problematic.

## Extracellular Vesicles and their Cargo

One of the most recently identified breast milk components that may alter the intestinal immune response and subsequent establishment of the microbiota are the extracellular vesicles (EV) that contain a rich protein cargo, capable of influencing the local immune response to bacterial challenge (111, 112). Hence, the discovery 10 years ago that human breast milk contains abundant EVs has garnered a lot of attention in the field (113). EVs contain a diverse cargo, including mRNA, miRNA, and cytosolic and membrane proteins and have been demonstrated to be intricately involved in cell–cell signaling. EVs include exosomes, which form through the endosomal pathway, and are released from cells following fusion of multivesicular endosomes with the plasma membrane. The larger (0.1–2 µM), more heterogenous microvesicles are formed through direct blebbing from the cell plasma membrane. It is important to note that many breast milk studies use the term “exosomes,” but do not separate exosomes from other vesicles, neither conceptually nor physically. Unless the isolation procedure takes advantage of exosomes’ known size or flotation density (e.g., through sucrose gradients, or size exclusion chromatography) or their known markers (i.e., by immunomagnetic isolation, e.g., anti-tetraspanin beads), isolated vesicles cannot be definitively described as exosomes. Both ultracentrifugation and PEG-based reagents such as Exoquick™, commonly used in breast milk studies to date, will pellet other vesicles as well as non-vesicular proteins, including RNA-binding proteins. Studies that use these methods have still revealed exciting potential for breast milk EVs, in terms of biomarkers, or biological activity *in vivo*. The Nolte-‘t Hoen group have published a useful study that compares EV isolation methods from breast milk (114).

Breast milk EVs contain RNA (115), miRNA, and long non-coding RNA (116). Several studies that profiled miRNA in breast milk exosomes found enrichment in multiple biological functions, including regulation of actin cytoskeleton, glycolysis/gluconeogenesis, aminoacyl-tRNA biosynthesis, pentose phosphate pathway, galactose metabolism, and fatty acid biosynthesis, as well as a wide range of immunological pathways (117–120). Likewise, proteomic analysis of human breast milk EVs revealed that the majority of proteins mapped to immune cell origin (121). Interestingly, a large number of these proteins had not been previously identified in human breast milk, demonstrating that exploration of EV cargoes may reveal novel biomarkers and functional pathways for further investigation. Exosomes in bovine milk are also enriched in proteins involved in immune response and growth (122).

Exosomes can mediate delivery of novel functional miRNA and mRNA to recipient cells (123). Whether miRNAs in breast milk exosomes are functional in the human digestive system is still relatively unknown; some studies show that exosomes protect miRNAs from digestion (118, 124), while others show that miRNAs are degraded by intestinal contents (125). Certainly, breast milk mRNAs and miRNAs can be taken up by cells and elicit functional effects *in vitro*, suggesting the exciting possibility that they may be able to alter protein expression at the neonatal mucosal surface, impacting on the development of the infant’s immune system. These functional effects demonstrated thus far include inhibition of *in vitro* T cell cytokine production and boosting regulatory T cells (113) and inhibition of HIV-1 infection of dendritic cells (126). Liao et al. also recently demonstrated that milk-derived EVs enter human intestinal crypt-like cells, with some localization to the cell nucleus; thus, this is a potential mechanism for delivery of immunoregulatory genetic material from mother-to-infant cells (127). Administration of breast milk exosomes increases intestinal epithelial proliferation in both pigs (128) and rats (129), suggesting that they also have the potential to promote normal intestinal development and function in neonates. In addition to acting in the intestinal tract, EVs could potentially exert effects in the oropharynx and nasopharynx. Thus, breast milk EVs could alter the neonatal immune response to oral vaccines, respiratory pathogens and colonization.

Extracellular vesicles also have the potential to modulate the host–microbe interaction. Epithelial and immune cell responses to gut microbes *Lactobacillus* or *Bifidobacterium* are modulated in the presence of EVs from serum (111). These EV enhance aggregation and phagocytosis of bacteria, as well as modulating TLR responses. Whether these activities are also performed by breast milk EVs is not known. As well as human milk, EVs also have been detected in porcine (128), bovine (122), and murine (125) milk, enabling the use of animal models to explore this phenomenon, as well as raising the possibility of there being cross-kingdom cell–cell communication *via* unpasteurized milk. Studies in mice have identified that the absence of EVs decreases the diversity of the pup intestinal microbiome (130). Human studies of the role of exosomes and their cargo in modulating infant intestinal microbiome are limited. However, Kosaka et al. identified miRNA associated with immune regulation within exosomes in breast milk that are particularly abundant in the first 6 months of life, when the neonatal mucosal immune system is developing (118). Recent work investigating the role of miRNA in EVs in the ProPACT trial demonstrated an array of miRNA in human milk that differed between mothers given probiotics and those given placebo but no significant differences in atopy outcomes (131).

The few studies of exosomes in breast milk to date have often been cross-sectional (116), and there is only one study of exosomes in human colostrum (113); milk that is delivered at a key stage for early immune priming. A study of bovine exosomes shows that the immunomodulatory protein cargo changes temporally during lactation (122); thus, detailed exploration of human breast milk EV cargo across the course of lactation could yield data that are highly relevant to the development of the neonatal immune system. Isolation of exosomes from breast milk to investigate the miRNA and protein cargo that could be delivered to the infant mucosa would offer novel insight into potential delivery mechanisms for drugs with intestinal immunomodulatory factors (132). Furthermore, improved knowledge of the stability and functionality of EV cargoes *in vivo* is vital for our understanding of how breast milk improves neonatal health and immunity.

## Future Directions

### Breast Milk Microbiota

Many unanswered questions regarding the microbiome need further exploration. We need more studies to define the mechanism by which the microbiota impact immune development and how dysbiosis leads to gut inflammation. Greater comprehension beyond the community profile to elucidate function and metabolites produced by the microbes is integral to utilizing these pathways to improve health or alter disease outcomes. If an enteromammary pathway is confirmed, we could exert a positive influence on infant health by modulating the maternal gut microbiota. Breast milk studies to date have mainly focused on the bacterial component. We also need to further understand how the milk virome and mycobiome influence infant gut health.

### Breast Milk HMOs

Further questions surround the HMOs, namely, functions of individual HMO and synthesis of HMO in the laboratory for nutrition supplementation; manipulation of HMO expression; and their delivery to establish a healthy microbiome. It is also possible that early intervention (within the first few days of life) is required for such therapies to be successful.

### Breast Milk EVs

For future studies of breast milk EVs, including exosomes, it is key to ensure that the correct nomenclature is utilized, based on the isolation methods used. Utilizing the guidelines of the International Society for Extracellular Vesicles (114) and reporting isolation methods through the new EV-TRACK database (133) will greatly aid the field of breast milk EVs. Apoptotic bodies have been seen as something to deplete in breast milk studies to date. Nothing is known about their cargo nor their function in breast milk, but they could play an important biological function in the neonate, as seen in other fields. We also lack detailed understanding of how breast milk EVs change over the course of lactation in humans. We need to understand better how breast milk EVs survive *in vivo* in the oropharynx, nasopharynx, and the gut, where their delivery would be critical. Finally, a human model of EV interaction with the neonatal microbiome would also give critical insight into possible mechanisms that could be harnessed to protect infants from disease and aid intestinal immune development in term and preterm infants alike.

### Summary

Future research studies should aim for enrollment of mother–infant pairs, large sample sizes, and longitudinal sample collections and include a diverse population to further elucidate variability in the breast milk microbiome, HMOs, and EVs on infant health outcomes. Studies should employ metagenomics, metatranscriptomics, and metabolomics approaches to understand the complete taxonomical, functional, and metabolic profile and create a more accurate picture of the breast milk contribution to infant health. Studies of the breast milk virome and fungome are warranted. Furthermore, ensuring that a repository of maternal and infant samples is kept for future research is useful in determining the long-term health implications of the gut microbiome present during the critical window. A repository can also present the opportunity to study the multigenerational transmission of microbes, HMOs, and EVs, facilitating a comprehensive understanding of the dynamics of the mother’s contribution to the infant immune system.

## Author Contributions

PP conceived and designed the manuscript. KLD, BH, AB, and PP contributed to the drafting and critical revision of this manuscript. All authors approved the final copy of the manuscript.

## Conflict of Interest Statement

KLD has received funding for investigator led initiatives from GSK and Pfizer. PP receives funding from AstraZeneca and MedImmune for unrelated studies. All other authors declare no conflicts of interest.
